# Accelerated Sorption Diffusion for Cu(II) Retention by Anchorage of Nano-zirconium Dioxide onto Highly charged Polystyrene Material

**DOI:** 10.1038/srep10646

**Published:** 2015-07-17

**Authors:** Qingrui Zhang, Qing Du, Tifeng Jiao, Jie Teng, Qina Sun, Qiuming Peng, Xinqing Chen, Faming Gao

**Affiliations:** 1 State Key Laboratory of Metastable Materials Science and Technology, Yanshan University, Qinhuangdao 066004, PR China; 2 Hebei Key Laboratory of Applied Chemistry, School of Environmental and Chemical Engineering, Yanshan University, Qinhuangdao 066004, PR China; 3 Shanghai Advanced Research Institute, Chinese Academy of Sciences, Pudong Shanghai 201203, PR China

## Abstract

The development of nanocomposite with strong adsorption ability exhibits great potential applications for environmental remediation. However, the pore blocking in preparation frequently constrains sorption diffusion, resulting in low utilization efficiency. Here we synthesized a new nano-ZrO_2_/Polystyrene (NZO-PS) material tailored with a specific fixed SO_3_-Na group to enhance Cu(II) removal. The NZO-PS exhibits efficient Cu(II) sequestration in a wide pH range (3.0–6.5) and preferential sorption performances. The efficient kinetic behavior and column applicability suggest the blocked pore channel is not a matter when presence of negatively charged moiety, which accelerates Cu(II) sorption diffusion and enrichment toward target active site. Moreover, the exhausted NZO-PS can be readily regenerated through HCl-NaCl binary solution. The preparation route can be extended to synthesize other functional composited materials. Simultaneously, the characteristics of simplicity, high-yield and regeneration provide some promising industrial merits.

Heavy metal pollution in waters is a worldwide environmental issue due to their toxicity and carcinogenicity, some toxic metals are dangerous even at trace concentration^1^. Among the available methods, adsorption is one of the most promising strategies for trace heavy metal retention^2^,^3^,^4^,^5^,^6^,^7^. Therefore, developing advanced functional materials for target pollutants have been actively conducted. In recent decade, numerous of nanoscale materials –nanosized zirconium oxides^8^,^9^,^10^, flower-like MgO^11^,^12^ and urchin-like FeOOH^13^- are exploited for heavy metal and environmental remediation, which exhibit superior sorption capacities and selectivity^14^,^15^,^16^,^17^. Such outstanding performances are mainly dependent on their unique morphologies, enhanced reactive activity and the strong sorption affinities^18^. However, the difficulties in solid-liquid separation and large hydraulic resistance might be the severe challenges, thus the development of nano-adsorbent in engineering merit is of strategic importance to heavy metal capture.

Recently, a large family of hybrid nanomaterial with excellent environmental properties has stimulated research enthusiasm. Briefly, the hybrid nanomaterials are fabricated by impregnating nanoparticles onto various porous carriers (e.g. granular activated carbon^19^, silica^20^, zeolite^21^,^22^ and macroporous polymer^23^,^24^). The obtained composite can combine the excellent flow characteristics of the porous supports and the specific affinities between nanoscale particles and heavy metal. Particularly, the template structures of matrix with nanopores can greatly improve the morphologies and reduce nanoparticle sizes^25^,^26^. Therefore, such hybrid fabrication has shed some lights on further engineering nanoparticles.

Now, most researchers always pay more attentions to the astonished morphology and controllable preparation^27^,^28^. Nevertheless, the well-defined morphology with high sorption capacities aren’t always the exhibition of satisfactory performances in application views^29^,^30^ Specifically, the serious pore blocking in the anchorage of nanoparticle always leads to poor sorption diffusion, which will bring about the low sorption efficiency in high-speed flow application^31^,^32^ Viewing the classical intraparticle-diffusion theory^33^,^34^, the sorption permeation is mainly depended on the pore channel structure and a narrow entry interspace can significantly inhibit the accessibility of target heavy metals. Therefore, how to break through the diffusion barriers of pore structures is a severe problem for resolving.

As well known, highly charged unites can achieve the efficient ion directional diffusion by external electric fields, e.g. desalination by electrodialysis methods^35^,^36^,^37^. Therefore, we assume that the applying suitable charged surroundings within matrix interspace possibly accelerates the ion-diffusion toward available active sites. Here, we reported a new polystyrene/nano-ZrO_2_ composite (denoted as NZO-PS) modified with the sulfonate (-SO_3_^−^Na) groups for Cu(II) sequestrations. The immobilized negatively charged groups can attract the target metals by strong electrostatic forces, boost the ion-transport and further improve the sorption utilization of nanoparticles. The representative toxic metal Cu(II) is selected as target pollutant and series batch and column tests are performed to evaluate the possible sorption behaviors and diffusion mechanism.

## Results

### Characteristics of NZO-PS

**Figure 1:**
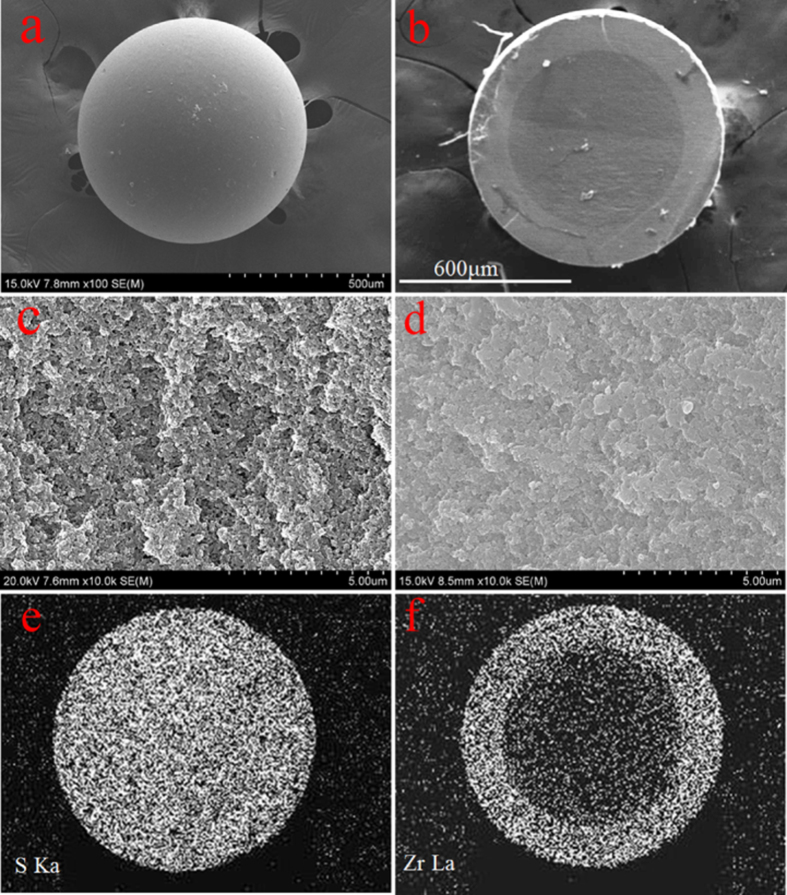
SEM characterization of NZO-PS: (**a**) SEM of the spherical NZO-PS bead; (**b**) the hemisphere profile of NZO-PS (**c**) SEM of PS inner surfaces (**d**) SEM of of NZO-PS inner surfaces; (**e**) the cross-section S distribution (**f**) the cross-section Zr distribution of NZO-PS by SEM-EDS.

The detailed structure parameters of obtained NZO-PS are shown in Table S1 and systematic characterizations are illustrated in Fig. 1. Figure 1a suggests that NZO-PS is the spherical beads with sizes of approximately 800 μm. The different SEM micrographs (Fig. 1c,d) of hemisphere profile indicate that the ZrO_2_ particles are well incorporated into the inner surface regions and block parts of cross-linked pores. The EDS profile scanning analysis (Fig. 1e,f) reveals the entrapped ZrO_2_ particles are uniformly dispersed in a ring-like region.

**Figure 2:**
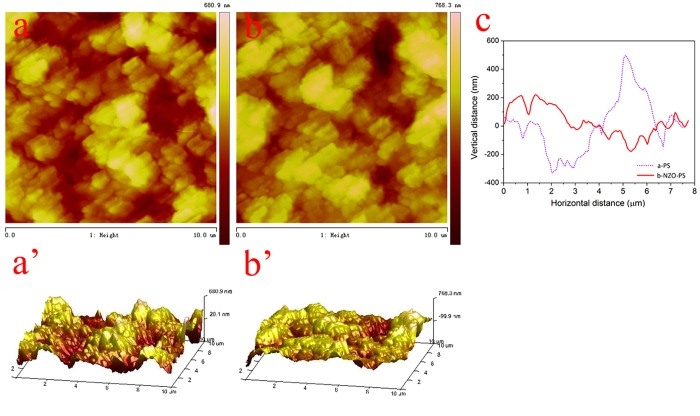
AFM analysis of NZO-PS; (**a**) AFM line profile analysis of PS; (**b**) AFM line profile analysis of ZrP-MPN; (a) AFM 3D analysis onto the inner surface of PS; (b) AFM 3D analysis onto the inner surface of NZO-PS; (**c**) surface height variations comparison before and after ZrO_2_ loadings.

Moreover, AFM analyses (Fig. 2) further gain new observations into the different morphologies before and after nano-ZrO2 incorporation. It can be detected that the inner surfaces profile of matrix PS displays outstanding height difference with a vertical height approximate ±400 μm, indicating the broad cross-linked macroporous structures. Comparatively, for NZO-PS, the visible highlight regions and smooth surface height variations (approximate 20–100 nm) further prove the successful implantation of ZrO2 particles and the possible resulting pore blocking

**Figure 3:**
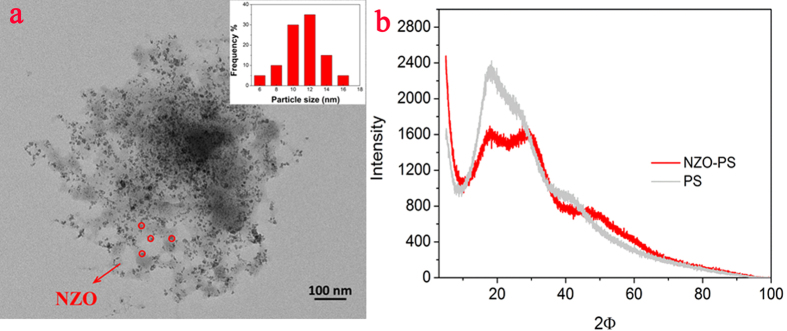
(**a**) The TEM characterization of NZO-PS; (**b**) XRD patterns of the obtained NZO-PS and matrix PS.

BET results (Table S1) show that the ZrO_2_ incorporation results in a understandable drop in pore volume and average pore diameter. Interestingly, a slightly increase in BET surface areas from 14.8 to 18.3 m^2^/g is possibly ascribed to the presence of nanostructure ZrO_2_ particles with higher surface area. TEM image (Fig. 3a) of NZO-PS further reveals that the implanted ZrO_2_ particles exhibit outstanding monodispersity with sizes ranging from 8 to 14 nm. Such well-defined morphology can be partly attributed to the cross-linked nanoporous template effects as well as the highly charged surroundings from the -SO_3_^−^Na^+^ of matrix^38^,^39^. X-ray diffraction investigation proves the amorphous or low crystalline pattern of the encapsulated nano-ZrO_2_ (Fig. 3b) by observing the high background noise and broad peaks. Considering the mild synthesis conditions, thus, it is readily to prepare the amorphous pattern. In facts, the amorphous ZrO_2_ particle is more beneficial to sequestration of Cu(II) ions and the highly crystalline forms will constrain the Cu(II) accessibility by the crystal lattice match and restraint, which has been confirmed in our previous study^40^.

### The effects of solution pH on adsorption

**Figure 4:**
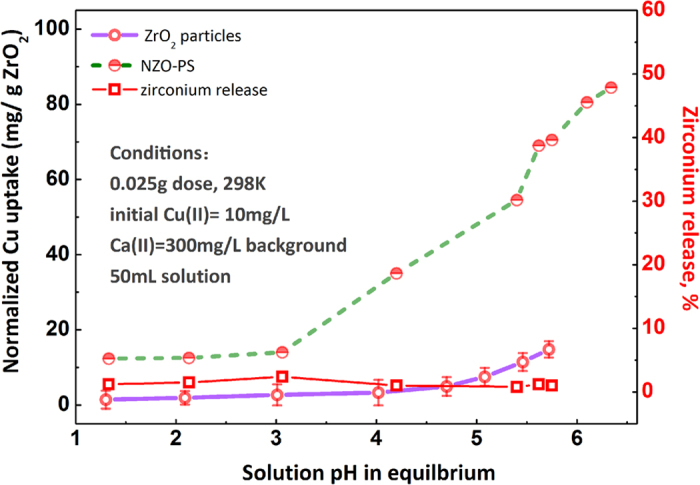
Effect of solution pH on the uptake of Cu(II) ions onto NZO-PS and ZrO_2_ at 298 K.

Series batch tests for adsorption performances toward toxic Cu(II) ions were conducted using conventional bottle-point method. The Cu(II) uptake is a pH-dependent process (Fig. 4) for both purified ZrO_2_ and composite and calcium ions are also introduced for screening the potential adsorption by SO_3_Na groups. Especially notice that the ZrO_2_ particles exhibit negligible Cu(II) uptake with solution conditions until pHs > 5.0, indicating its limited applicability to acidic surroundings. Comparatively, for NZO-PS, the encapsulated ZrO_2_ particles display the considerable sorption performances at broad applied pH ranges (pH > 3). Such distinct appearance can be ascribed to the unique surface properties of NZO-PS. The potential proton buffering effects from sulfonic acid groups (SO_3_Na) and inherent high cross-linking structures^41^ can lead to the acidic-basic variance of inner/outer regions. Besides, the immobilized nano-ZrO_2_ with large surface areas and active sites, also imparts more powerful sorption affinities toward Cu (II) removal than the bulk one^14^,^17^ and the trace zirconium release further demonstrate its stability and applicability.

### Common competition influences on adsorption

**Figure 5:**
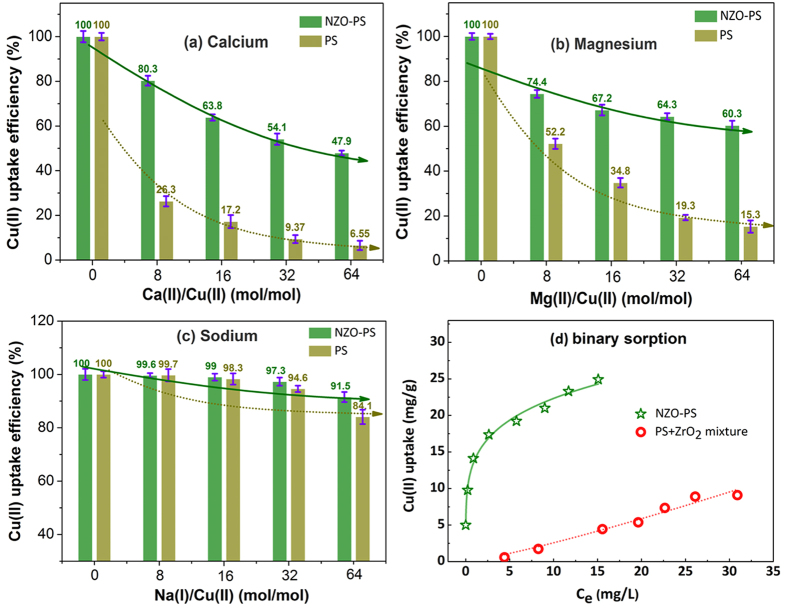
Cu(II) sorption competitive comparison of NZO-PS and its host material PS (**a**) Ca(II) ions interfere; (**b**) Mg(II) ions interfere; (**c**) Na(I) ions interfere; (**d**) comparison of sorption behaviors onto NZO-PS and its corresponding binary mixture ZrO2 + PS (For (a−c) 1 g/L sorbent, initial Cu(II) = 0.25 mM, pH = 5.2−5.8; (d) adsorbent: 0.1 g NZO-PS and 0.088 g PS + 0.012 g ZrO_2_ sorbent, 500 mg/L background calcium ions).

In general, the common cations, particularly for Ca(II) Mg(II) and Na(I), are always ubiquitous in the metal-contaminated waters, the sorption interferences may be a main adverse influence due to its high contents and the same positive-charge. Therefore, it is significant to validate the underling sorption selectivity onto the given NZO-PS. Figure 5a–c shows that both NZO-PS and PS display the decreasing sorption tendency with bivalent Ca(II)/Mg(II) except for Na(I) additions continuously. Comparatively, the composite NZO-PS still exhibits distinguished Cu(II) uptake performances. Of particular note, the Cu(II) removal is slightly influenced with above 16 times Ca(II) additions, while the Cu(II) uptake onto PS brings out a dramatic decrease (near to zero) at the same conditions. In general, the sulfonate moiety binding onto PS always presents nonspecific electrostatic forces toward Cu(II) exchange and the high level of common cations additions will result in serious sorption competition. Whereas, for NZO-PS, the encapsulated ZrO_2_ nanoparticles can exhibit powerful inner-sphere surface complexation^42^,^43^, i.e. the monodentate and bidentate chelation. Such specific affinities will greatly enhance the Cu(II) adsorption onto NZO-PS, which has been demonstrated by EXAFS and XPS analysis^44^,^45^. Moreover, considering the possible coexistence of other heavy metals and strong competition in wastewaters, three common heavy metals (Zn(II), Ni(II), Cd(II)) were selected to evaluate the potential sorption influences and the results were shown in Figure S2. Similar to the alkaline metals additions, NZO-PS and its matrix PS also display decreasing sorption performances, particular for Ni(II) and Cd(II). Comparatively, NZO-PS still shows preferential Cu(II) sequestrations, further indicating its strong affinities and applicability. Additionally, high contents of common anions were also involved to examine the possible sorption effects (Figure S3). As is expected, the negligible Cu(II) sorption variations in absent or present anions (NO_3_^−^, Cl^−^, SO_4_^−^) also demonstrate that anions might be the weak competitors for Cu(II) uptake.

**Figure 6:**
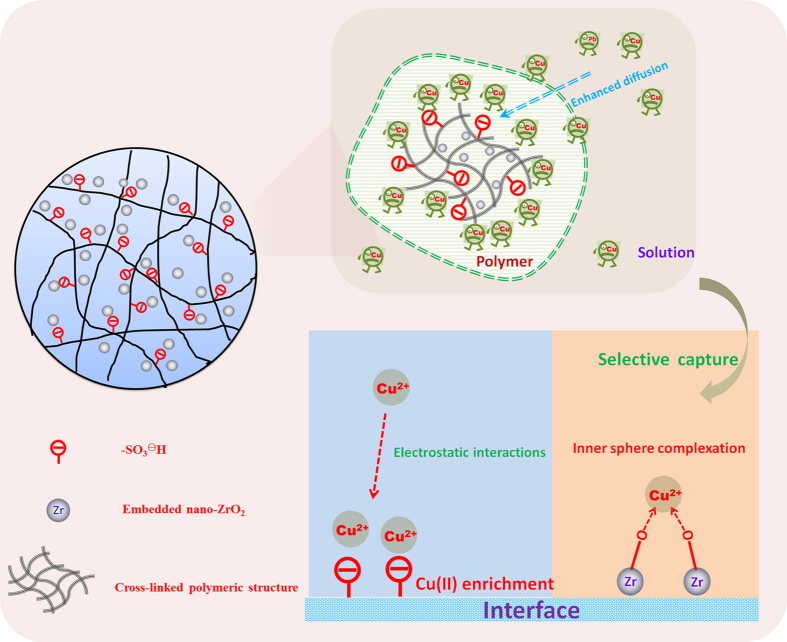
The schematic structure and sorption mechanism of the resulting NZO-PS.

Furthermore, the composite NZO-PS and its corresponding composition ZrO_2_ powders and PS beads mixture were also investigated for Cu(II) elimination. As depicted in Fig. 5d, NZO-PS reveals more favorable sorption behaviors than its binary mixture, which further proves the importance of hybridizing nanomaterial fabrication. The preferential sorption onto NZO-PS can be ascribed to the following aspects. (1) The negative-charged SO_3_^−^Na moiety within matrix can accelerate the target Cu(II) ions diffusion, and then form high concentration of Cu(II) ions enrichment within the nanopores phase of NZO-PS. Such acceleration mechanism is derived from the classical “Donnan membrane effects”^46^ (2) The crosslinking polymeric chains of matrix can also facilitate the formation of immobilized nano-ZrO_2_ particles^47^. Therefore, the collaborative efficient sequestration in the reactive interface is achieved though the highly charged SO_3_^−^Na transport enhancement and selective Cu(II) uptake onto embedded nano-ZrO_2_. A colourful schematic structure and sorption mechanism of NZO-PS are shown in Fig. 6.

To further elucidate the sorption priority, the selective distribution constant K_d_ (in L/g) was determined as the following equation:


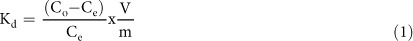


where C_0_ is the initial Cu(II) concentrations of the solute, C_e_ is the Cu(II) effluents in equilibrium, V represents the volume of the solute, and m is the mass of the material. Evidently, the substantially larger K_d_ values of NZO-PS further prove the strong affinities and favorable selectivity (Table S2).

**Figure 7:**
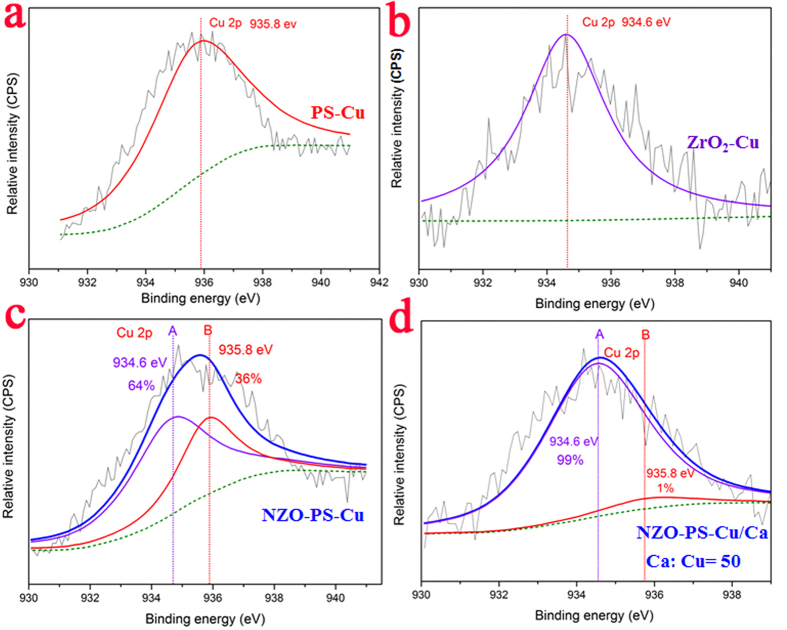
High-resolution XPS spectra of NZO-PS. **a**) Cu 2P spectra of Cu(II) loaded PS samples; **b**) Cu 2P spectra of Cu(II) loaded purified ZrO_2_ samples; **c**) Cu 2P spectra of Cu(II) loaded NZO- PS samples; d) Cu 2P spectra of Cu(II) loaded NZO- PS samples in presence of high level of Ca(II) ions competition.

To gain further insight into the sorption behaviors, Fourier Transform Infrared Spectroscopy (FT-IR), X-ray Photoelectron Spectroscopy (XPS) and Zeta Potential Plus analysis were conducted to obtain the possible mechanism for selective Cu(II) uptake. The distinct IR absorption peaks at 1183 cm^−1^ and 1128 cm^−1^ indicate the presence of SO_3_^−^H groups (Figure S4-a). In general, the Cu(II) uptake by SO_3_^−^Na are nonspecific affinities, driven by electrostatic attraction and the strongly specific adsorption is mainly ascribed to the existence of nano-ZrO_2._ The sharp peaks (Figure S4-b) at approximate 502 cm^−1^ are assigned to Zr-O bond, while Cu(II) ion uptake induced to the apparent band shifts from 502 cm^−1^ to 505 cm^−1^, suggesting the formation of strong sorption affinities. XPS investigation further demonstrate the different sorption behaviors. Figure 7a reflects the Cu 2 p binding energy of 935.8 eV by SO_3_-Cu, while the distinct Cu 2 p binding energy peak at 934.6 eV is ascertained by Cu(II) loaded ZrO_2_ particles, the apparent shifts of 1.2 eV to a lower energy level reveal the formation of the specific affinity between Cu(II) and ZrO_2_ (Fig. 7b). Figure 7c displays the representative Cu 2 p binding energy state of Cu(II) uptake onto NZO-PS, the expanded full width at half maximum (FWHM) implies the presence of different Cu-complex substances. Based on the binding energy of diverse Cu(II) species or sorption sites, the Cu 2 p spectra are divided into two peaks corresponding to SO_3_-Cu (935.8 eV) and Zr-O-Cu(934.6 eV). As for Cu(II) loaded NZO-PS, the area fractions of SO_3_-Cu (935.8 eV) and Zr-O-Cu(934.6 eV) are occupied around 36.0% and 64.0% respectively. However, 50 times of Ca(II) addition brings about distinguished peak areas variations with nearly 1% SO_3_-Cu and 99% Zr-O-Cu respectively (Fig. 7d). Such results indicates that nano-ZrO_2_ can endow the strong sorption selectivity toward Cu(II) removal, and SO_3_^−^Na can only impart negligible Cu(II) adsorption performances at electrolytic backgrounds. Moreover, the evidence of highly negatively charges onto SO_3_^−^Na for diffusion enhancement is also proved by Zeta Potentials analysis. Figure S5 depicts the zeta potential variations at wide pH conditions onto NZO-PS and purified ZrO_2_ particles. Interestingly, the composite NZO-PS exhibits negatively large zeta potentials at broad pH ranges (2.0–13.0) and the surface charges of encapsulated ZrO_2_ and H^+^ additions will be negligible as compared to the highly negative sulfonate groups of host. Therefore, the diffusion enhancement and enrichment toward Cu(II) will be available at wide pH conditions. However, Considering the pH_zpc_ = 5.3 of ZrO_2_ particles, it is believed that the favorable sorption pH ranges from 5.3 to 6.2, which coincides with the solution pH effect results.

### Sorption kinetic evaluation and sorption isotherms

**Figure 8:**
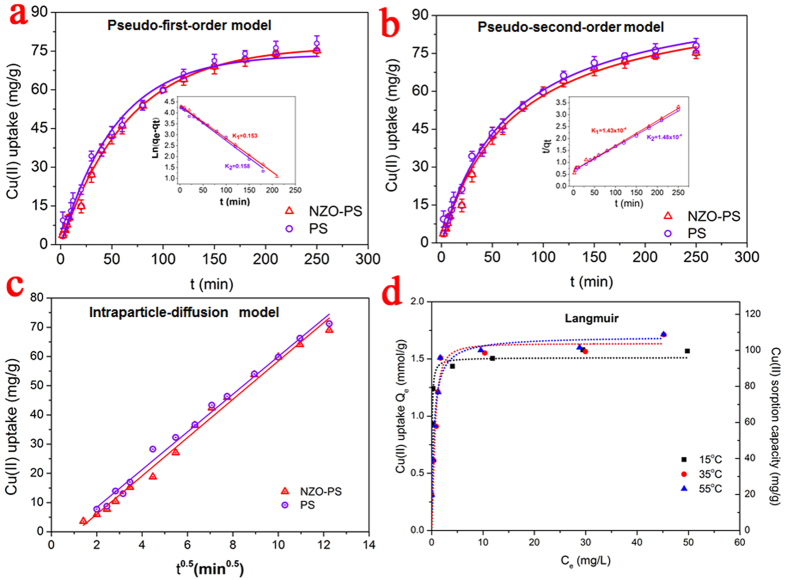
Adsorption kinetics of Cu(II) ions onto NZO-PS and its matrix PS at 298 K. (**a**) the pseudo-first-order fitting (a) the line fitting for K values; (b) the pseudo-second-order fitting (**b**) the line fitting for K values; (**c**) the intraparticle diffusion model (sorbent: 0.2 g/L, initial Cu(II) 0.5 mM pH = 5.3–6.1); (d) sorption isotherms onto NZO-PS at different temperatures by Langmuir model fitting.

Sorption kinetic experiments were conducted to further gain new insight into the ions transport enhancement. Figure 8a–c illustrates the Cu(II) sorption kinetic curves of NZO-PS/PS and 180 min minutes are satisfactory enough to approach sorption equilibrium. It is significant to note that the similar sorption equilibrium time onto both sorbents suggests the potential sorption diffusion enhancement. In general, the ion permeation is greatly dependent on the pathway of spherically porous structure. A narrow channel usually reflects the poor diffusion property, resulting in the prolonged equilibration time and low utilization of sorbent. Thus, the blocking pore structures onto NZO-PS are usually expected to show the inefficient Cu(II) ion sorption diffusion. Whereas, the similar sorption kinetic behaviors onto NZO-PS suggest that the pore diffusion will not be the principal constraints and the surface groups (-SO_3_^−^Na) within matrix will grant more prominent roles by electrostatic enhancement.

To further verify this opinion, the pseudo-first/second -order kinetic and Intraparticle diffusion models are performed and the detailed formulas as follows^48^:

The pseudo-first-order model:


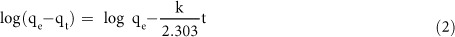


The pseudo-second-order model:


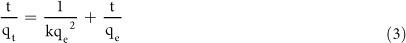


Intraparticle diffusion model:


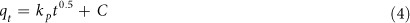


where q_t_ and q_e_ represent the amount of Cu(II) ions adsorbed (mg/g) in equilibrium and time t respectively, and k, k_p_ is the kinetic rate constant. Observably, the sorption process can be well described by the pseudo-second-order modeling (Fig. 8b Table S3) with a high correlation coefficient (R^2^) (>0.993). Besides, the excellent intraparticle diffusion fitting further indicates the intraparticle diffusion process. The similar K values of NZO-PS and PS further demonstrate the acceleration of ion transport.

Sorption isotherms were also performed at three different temperatures to examine the possible effects on Cu(II) uptake and the results are shown in Fig. 8d. Apparently, Cu(II) adsorption onto NZO-PS displays thermodynamics dependent process. Higher temperatures are favorable for Cu(II) uptake, which suggests the possible endothermic interactions with the maximum sorption capacities of approximate 108 mg/g. Additionally, the sorption behaviors onto NZO-PS are also described, according to the classical Langmuir, Freundlich isotherms as follows:

Langmuir model


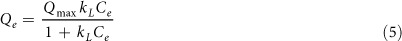


Freundlich model


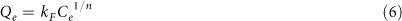


where C_e_ represents the Cu(II) concentrations at equilibrium, and Q_e_ is the corresponding adsorption capacity, Q_m_ is assigned to the maximum sorption capacity, k_L_ k_F_ and n are parameters.

The detailed fitting data are listed in Table S4. It is observed that Cu(II) uptake onto NZO-PS can be well described by Langmuir model and the maximum calculated capacity (108 mg/g) coincides with the sorption data in experiment. Besides, the sorption performances can also roughly compare with other Cu(II) adsorbents in literatures (Table S5). The large sorption capacity further verifies the significant application feasibility.

### The fixed-bed column application for Cu(II) retention

**Figure 9:**
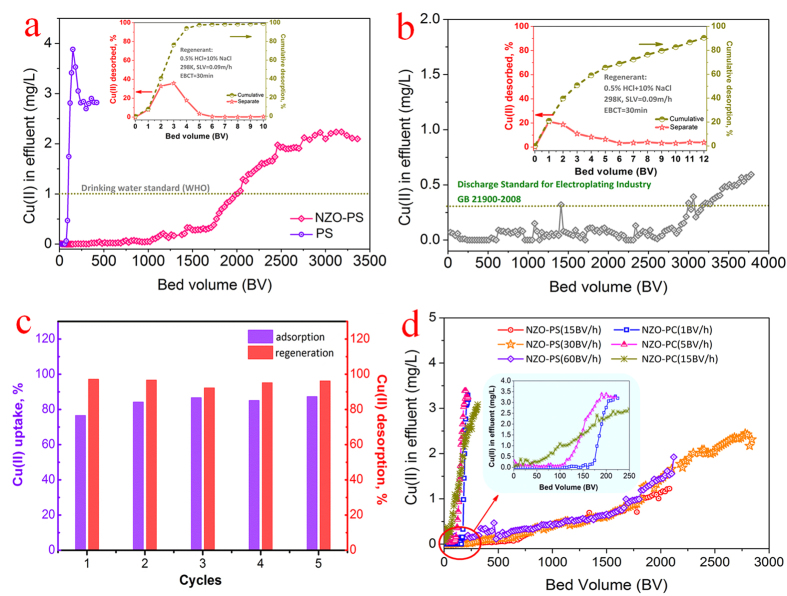
Comparison of breakthrough curves of Cu(II) uptake in fixed-bed columns. (**a**) The column adsorption curves of NZO-PS and PS; (adsorption: influent Cu(II) = 3 mg/L, Ca(II) = 200 mg/L, Mg(II) = Na(I) = 100 mg/L, pH = 5.5–6.2, SLV = 0.75 m/h, EBCT = 4 min); (**b**) the wastewater from electroplating industry (initial Cu(II) = 0.7–0.9 mg/L, SLV = 0.75 m/h, EBCT = 4 min); (**c**) batch sorption-degeneration tests onto NZO-PS at 303 K. (sorbent: 2 g/L, adsorption: Cu(II) = 50 mg/L, Ca(II) = 200 mg/L, Mg(II) = Na(I) = 100 mg/L, pH = 5.5 = 6.3; regenerant: 20 mL−0.5% HCl + 10% NaCl for 5 h at 298K) (**d**) influences of various feeding velocities on Cu(II) removal performance onto NZO-PS and NZO-PC.

High speed column tests were performed to evaluate the applicability of the given adsorbent. Figure 9a implies that NZO-PS displays efficient Cu(II) uptake performances in a superb fast fixed-bed system and the effluents can meet the drinking water standard recommended by WHO (1 mg/L) with 2000 bed volume (BV) treated capacities before significant breakthrough. Whereas, the inefficiency onto PS may be ascribed to the weak Cu(II) affinity and strong competition. Besides, the wastewater (detailed components in Table S6) from electroplating industry in Qinhuangdao, was also tested and the results of 3200 BV treated capacities further demonstrate the efficient applicability (Fig. 9b). Moreover, the exhausted NZO-PS can be readily regenerated in ten-bed volumes (efficiency: >90%) using 0.5% HCl + 10% NaCl binary solution. Batch sorption further verifies its efficient adsorption- regeneration stability with at least five recycled uses (Fig. 9c).

Additionally, such outstanding Cu(II) sorption behaviors are also greatly associated to its unique functional structure. i.e. Cu(II) ion diffusion enhancement though negative-charged SO_3_^-^Na groups. Note that a new composite (NZO-PC) modified with the neutral chloromethyl groups (-CH_2_Cl) is also involved for a reference and its detailed preparation (Figure S1), characterization (Figure S7) were shown in supporting information. Attractively, NZO-PS exhibits satisfactory Cu(II) sorption (Fig. 9d) by varying the high feeding speeds of 15 BV/h, 30 BV/h and 60 BV/h respectively. Whereas, the neutral NZO-PC displays inefficient Cu(II) uptake with the treated capacities of about 70 BV at the feeding velocity of 15 BV/h, further decreasing the feeding speed to 1 BV/h can moderately improve its sorption performances (170 BV treatments). Considering the similar nano-ZrO_2_ loadings and pore structures (Table S1), it is expected that the presence of negative-charged groups SO_3_^−^Na can remarkably increase the contact probability of target Cu(II) ions and available active sites, boost the potential Cu(II) sorption permeation, and further enhance the efficient Cu(II) sequestration onto NZO-PS.

Next, a simple box-model is assumed to further interpret the above sorption behaviors. We hypothesize that the whole sorption system is divided into two different phases by a hypothesis diffusion membrane. The left represents sorbent phase and the right is assigned to the solution.

In the case I: NZO-PC (Figure S8a), the 0.1 mM Cu(II) ions can move cross the diffusion membrane equally for equilibrium with the equation as follows:


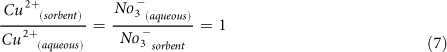


Nevertheless, for case II, (Figure S8b), the SO_3_^-^Na moieties are fixed onto NZO-PS and it is difficult to enter the right surroundings. Consequently, in sorbent phase, the extra-large charges will greatly attract the target Cu(II) ions diffusion and the enrichment environment (202 times calculated by Donnan principle^35^) in the left is realized at equilibrium, the detailed formula as follows:


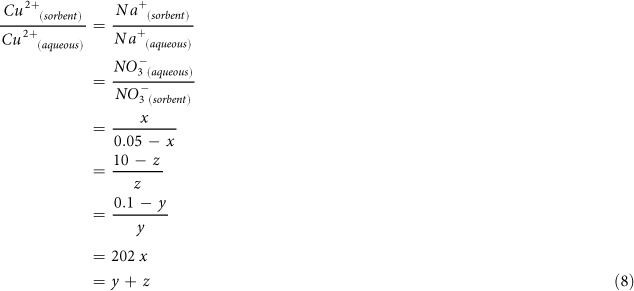


The above calculation suggests that the diffusion enhancement and enrichment proportion are greatly dependent on the group contents of R-SO_3_Na and Cu(II) concentrations, the high fixed-charges and trace feeding metallic ions can be preferential option. Such calculation results also agree with the results of column tests.

## Discussion

We offer a new route to break through the pore structure-dependent sorption diffusion process. Specifically, a new nano-ZrO_2_/polystyrene composite modified with a specific fixed–SO_3_^-^Na groups has been successfully fabricated for Cu(II) sequestration. The presence of negative-charged SO_3_^-^Na groups will enhance Cu(II) ions transport and enrichment by strong electrostatic force. Such functional design can overcome the potential application bottleneck for the blocked diffusion, and greatly promote the sorption performance of sorbent. As another point of view, such highly charged groups can increase the potential contact opportunities of nano-ZrO_2_ and target Cu(II) ions, which favors for trace heavy metals sequestrations.

As for the characterization of NZO-PS, the EDS analyses reflect the uniform modified S element and ring-like Zr distributions. Such phenomenon is interesting and the uniform coverage of sulfur element testifies the homogeneous sulfonation reactions for SO_3_^−^Na introduction. While, the incorporation of nano-ZrO_2_ may be a gradual diffusion process and the pore blocking in preparation will restrain zirconium salts precursor and strongly alkaline solutions diffusion into the center areas for active ZrO_2_ formation. Besides, the slightly increase of BET surface areas from 14.8 to 18.3 m^2^/g after immobilization can be possibly ascribed to the nanoscale sizes of ZrO_2_ particles, which imparts sufficient surface area and abundant activated sites for target heavy metal removal.

An interesting observations are the different pH behaviors for Cu(II) captures onto NZO-PS and ZrO_2_ powers. The favorable Cu(II) uptake is detected at pH = 3-6 onto NZO-PS, and solution pH > 5 for bulk ZrO_2_. Such phenomenon can be ascribed to the different H^+^ activities of inner pore and external solutions. Note that the strong crosslinking net structure of polymeric matrix can anchor the embedded nano-ZrO_2_ particles to play a significant role for the solution chemistry variations. In addition, the surface negatively charged sulfonate group (SO_3_Na) can further buffer the H^+^ accessibility by the formation of protonated SO_3_H. The results imply that NZO-PS can work more efficiently under weak acidic solution than the bulky ZrO_2_ particles, which extends the applicability of nano-ZrO_2_ by hybridization design.

In summary, we fabricate a new hybrid nanomaterial by impregnating nano-ZrO_2_ onto polymeric porous polystyrene modified with sulfonate group (SO_3_Na). The charge-surface functionalization route can possibly enhance the target Cu(II) diffusion and promote utilization of embedding nano-ZrO_2_ particles. In addition, the hybrid material also exhibit superior selectivity and fast kinetic behaviors and recycled sorption-regeneration properties. All the results demonstrate the highly charged functionalization design is a smart choice for heavy metal remediation. Meanwhile, this route can be extended to prepare other functional composite materials.

## Experimental Details

### Fabrication of the hybrid HZO-PS

**Figure 10:**
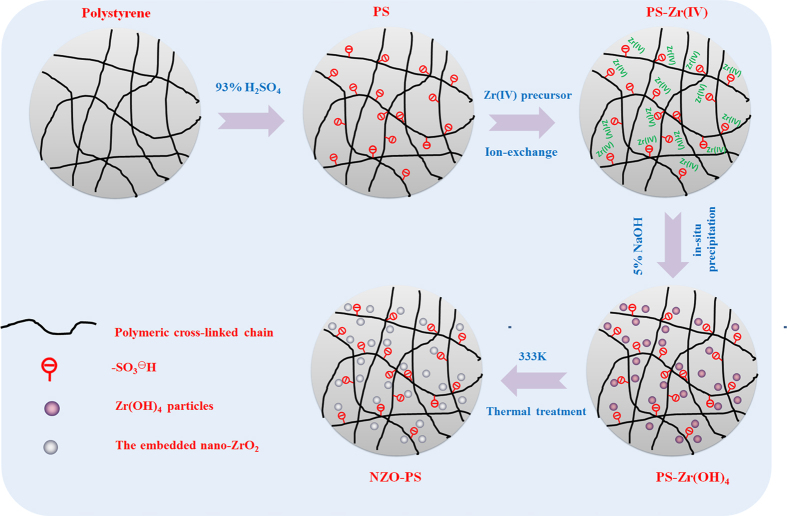
The simple preparation procedure of NZO-PS.

The fabrication of the resultant material was conducted (Fig. 10) as the following procedures. The polymeric polystyrene beads - prepared by the styrene and divinylbenzene suspension polymerization- were immersed into 93% sulfuric acid solution at 353–378 K for SO_3_H introduction^49^. Then the obtained sulfonated polystyrene beads (PS) will react with the Zr(IV) salts precursor by ion-exchange mechanism and obtained the intermediate PS-Zr(IV); afterward, 5% of NaOH solution was used as precipitant for in-situ formation of Zr(OH)_4_ in the inner pore regions of matrix. The framework nano-template and polymeric crosslinking chains could benefit to obtain the embedded nanoparticles. Finally, thermal treatment at 333 K was performed for the encapsulated nanoparticle immobilization and the encapsulated hydrated zirconium oxide nanocomposite NZO-PS was prepared.

### Batch Cu(II) Sorption tests

Series batch sorption experiments for adsorption performance towards Cu(II) uptake were performed by the bottle-point method. The effects of solution pH: 0.025 g of NZO-PS was introduced into 100 mL flasks containing 15 mg/L Cu(II) ions and 1% NaOH/HCl was used to adjust the desired pH values, the above bottles were then transferred into an incubator shaker at the constant temperature for above 20 h to ensure sorption equilibrium. Finally, the equilibrium pH and corresponding Cu(II) concentrations were determined. Competition tests were conducted by similar ways and the common cations -Ca(II), Mg(II), Na(II) ions- were added with different levels to evaluate the sorption selectivity toward Cu(II) ions. Kinetics tests were performed by sampling 1 mL-solution at various time intervals in a 1000 mL round-bottom flask containing 30 mg/L of Cu(II) ions and 0.2 g of NZO-PS beads. Ca(II) ions as competitor were also involved, if necessary, to estimate the potential ionic strength influence on kinetic permeation. Finally, kinetic data were calculated by determining the sample Cu(II) contents and its corresponding time. More experimental data were described in the captions and figures.

### The packed column experiments

The flow-adsorption column tests were performed in a small Plexiglas column (12 mm diameter and 150 mm length) equipped with water bath equipment. The peristaltic multi-channel pump was used for getting the desired liquid velocity. The water-swelling sorbent of NZO-PS/PS (5 mL) was packed in the separated columns. the known content of Cu(II) ion feedings for stimulating heavy metal contaminated waters and real electroplating wastewaters were used to validate the application feasibility of NZO-PS. The detailed hydrodynamic parameters and feeding conditions are shown in the figure captions. The regeneration were tested in the same column after adsorption, the binary 0.5%HCl + 10%NaCl mixture was used as the regenerant in a slow liquid velocity to ensure the fully stripping and the SLV and EBCT were equal to 0.05 m/h and 60 min, respectively. Next, the flow-speed test influences were then conducted to elucidate the potential kinetic diffusion mechanism and the composite material NZO-PC was involved for comparison. Note that in this tests, all the sorbents were packed with 3 mL of volumes to avoid the possible fluid resistance interference in the high-speed sorption operations.

### Characterizations

The Cu(II) contents were determined by a Shimadzu AA-6800 atomic absorption spectrometer equipped with deuterium background correction. The morphology of nanocomposite NZO-PS was taken by a field emission scanning electron microscope, FESEM (Hitachi S-4800, Japan). X-ray diffraction (XRD) patterns were performed on an XTRA X-ray diffractometer (Switzerland) and Cu Kα radiation (λ = 1.5418 Å) with a scan rate of 2^o^ s^−1^. AFM images were taken using multimode 8 scanning probe microscopes (Veeco Instrument Plainview, NY, USA) with silicon nitride cantilever probes. The pore structure parameters (surface areas, pore volume, average pore sizes) were determined by nitrogen sorption measurements using micrometrics ASAP 2020 (U.S.). TEM images were recorded using high-resolution transmission electron microscopy (HRTEM, JEM2010) equipped with a Gatan CCD camera working at accelerating voltage of 200 KV. The zeta potentials of adsorbents were measured in folded capillary cells using the Nano ZS90 zetasizer (Malvern Instruments, UK). XPS analysis of given samples were conducted with a spectrometer (UlVAC-PHI model 5000 Versa probe) and the results were corrected and fitted using a curve-fitting program (XPS-peak4.1), FT-IR spectral (Nexus870, USA) were examined with a pellet of powered potassium bromide and sorbent powders in 400–4000 cm^−1^.

## Additional Information

**How to cite this article**: Zhang, Q. *et al.* Accelerated Sorption Diffusion for Cu(II) Retention by Anchorage of Nano-zirconium Dioxide onto Highly charged Polystyrene Material. *Sci. Rep.* **5**, 10646; doi: 10.1038/srep10646 (2015).

## Supplementary Material

Supplementary Information
