# Supplementary material for: Accelerated Sorption Diffusion for Cu(II) Retention by Anchorage of Nano-zirconium Dioxide onto Highly charged Polystyrene Material
Source: Sci Rep. 2015 Jul 17;5:10646. doi: 10.1038/srep10646 (PMC4505334; doi:10.1038/srep10646)
Supplement: Supplementary Information [file srep10646-s1.doc]

Supporting Information

**Accelerated Sorption Diffusion for Cu(II) Retention by Anchorage of Nano-zirconium Dioxide onto Highly-charged Polystyrene Material**

Qingrui Zhang^a^, Qing Du^a^, Tifeng Jiao^a^*, Jie Teng^a^, Qina Sun ^a^, Qiuming Peng^b^, Xinqing Chen^c^* and Faming Gao^a^

^a^ Hebei Key Laboratory of Applied Chemistry, School of Environmental and Chemical Engineering, Yanshan University, Qinhuangdao 066004, PR China

^b^ State Key Laboratory of Metastable Materials Science and Technology Yanshan University

^c^ Shanghai Advanced Research Institute, Chinese Academy of Sciences, PR China

*To whom correspondence should be addressed (Tifeng Jiao/Xinqing Chen )

E-mail: [tfjiao@ysu.edu.cn/](mailto:tfjiao@ysu.edu.cn/) [chenxq@sari.ac.cn](mailto:chenxq@sari.ac.cn)

Tel: +86-335-8387-741

Fax: +86-335-8061-549

**Figure S1a** The preparation procedures illustration of NZO-PS

Step I：Sulfonation reactions for obtaining the PS beads

Step II：Nano-ZrO_2_ incorporation procedures onto PS


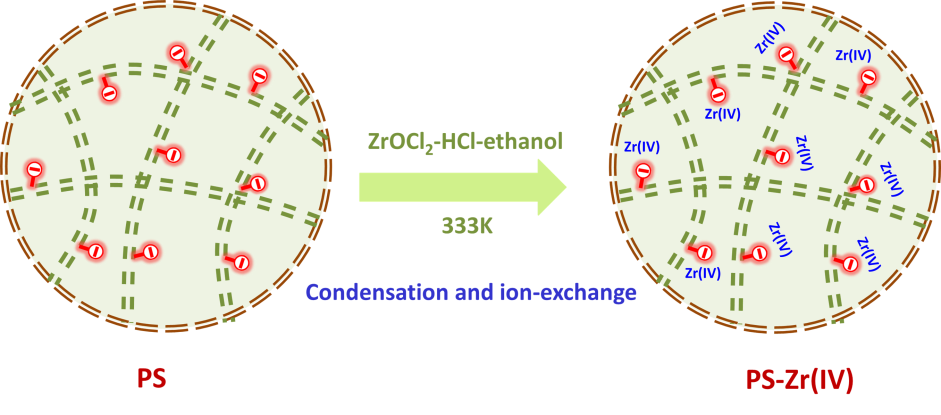


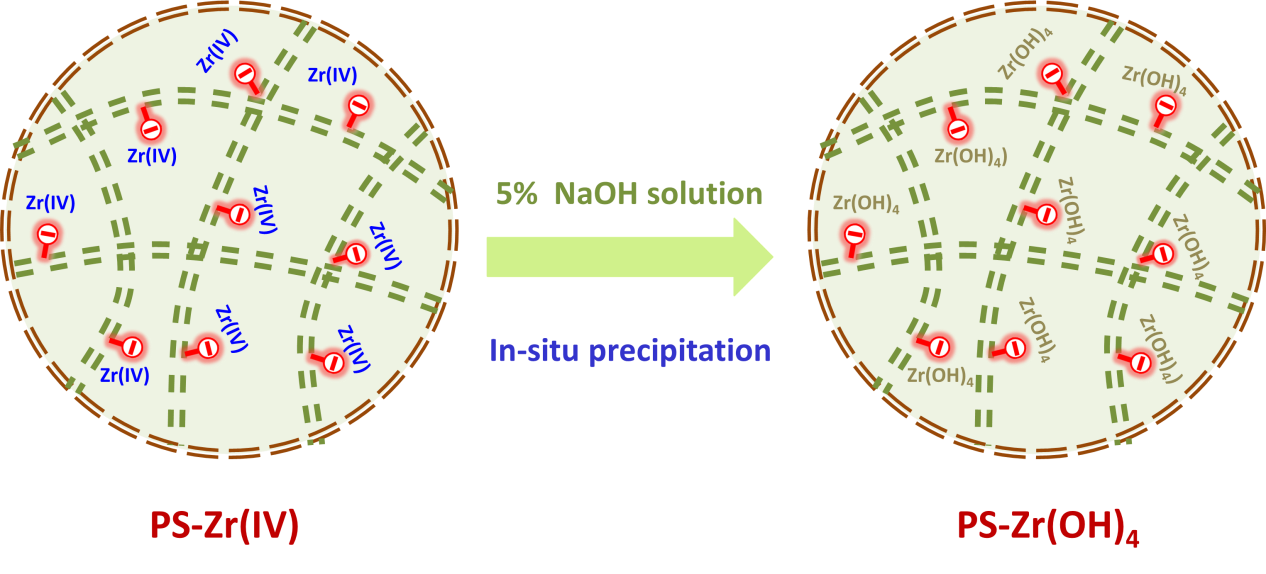


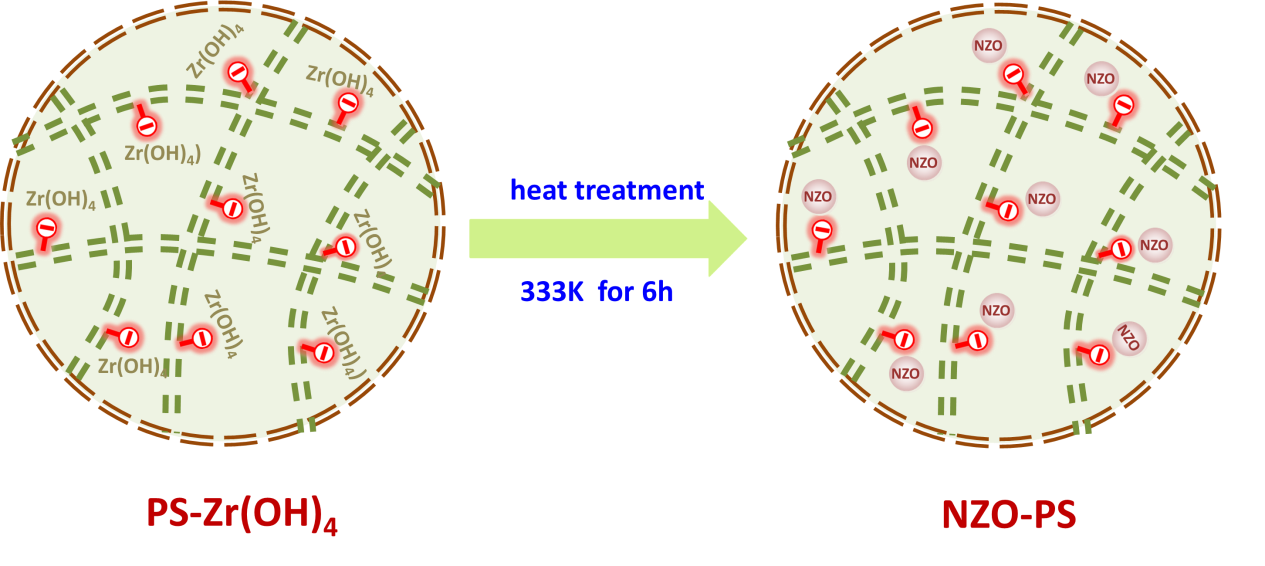


**Figure S1b** Fabrication of NZO-PC

Step I：Chloromethylation reactions for the PC preparation

Step II：Nano-ZrO_2_ incorporation procedures onto PC

The preparation methods were very similar to that for NZO-PS and the simple procedures as follows:


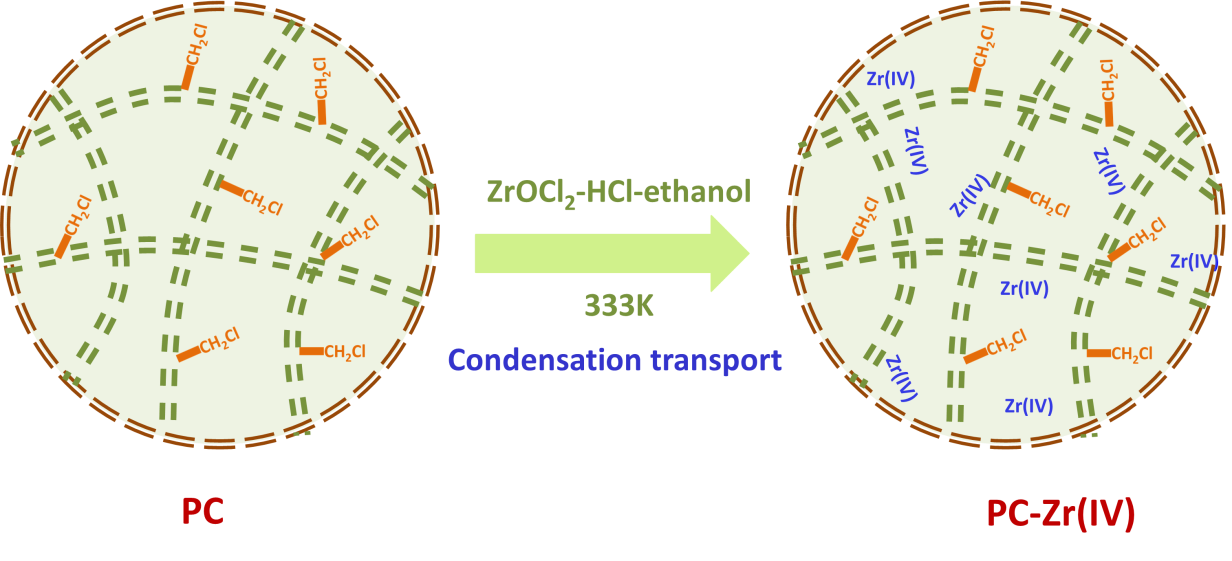


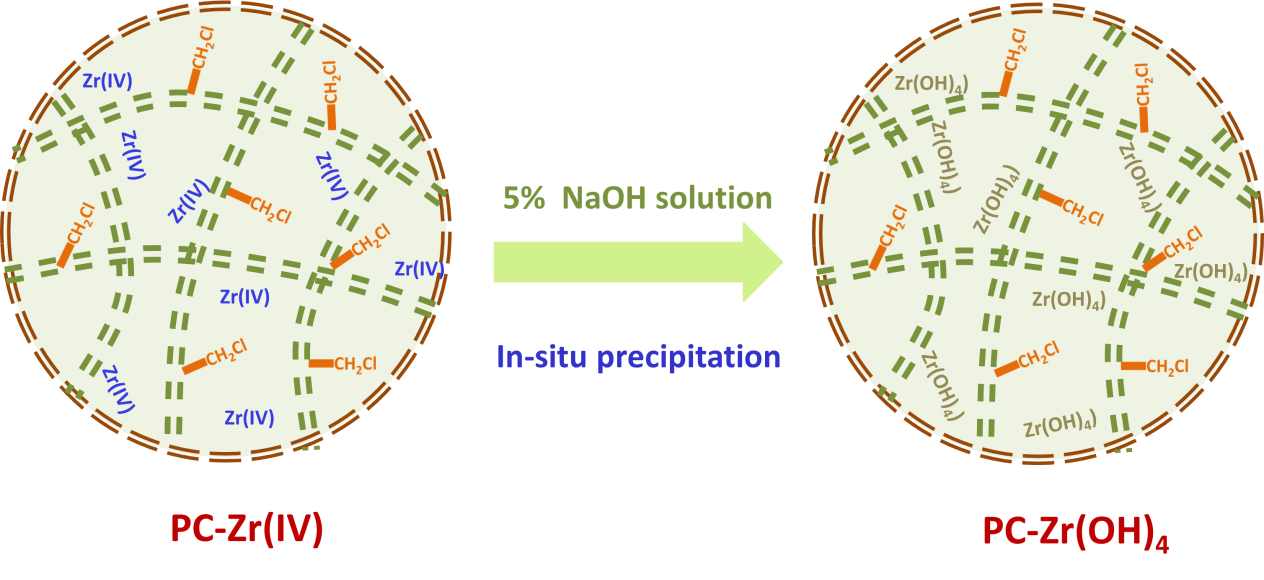


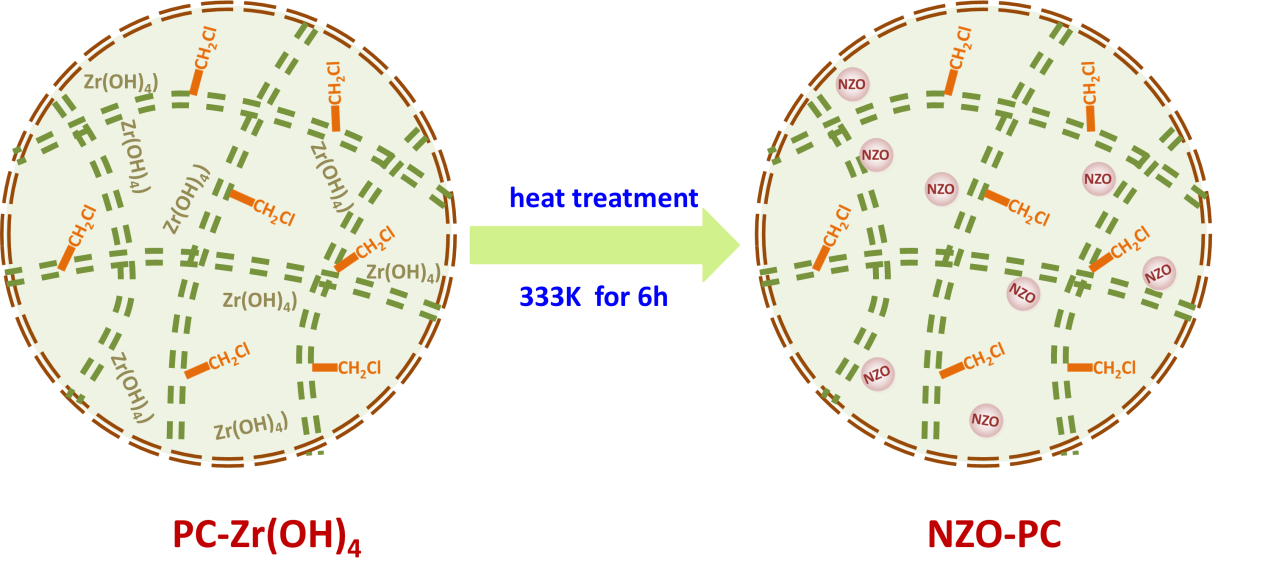


**Figure S2** Cu(II) sorption competitive comparison of NZO-PS and its host material PS (a) Zn(II) ions interfere; (b) Cd(II) ions interfere;(c) Ni(II) ions interfere;

**
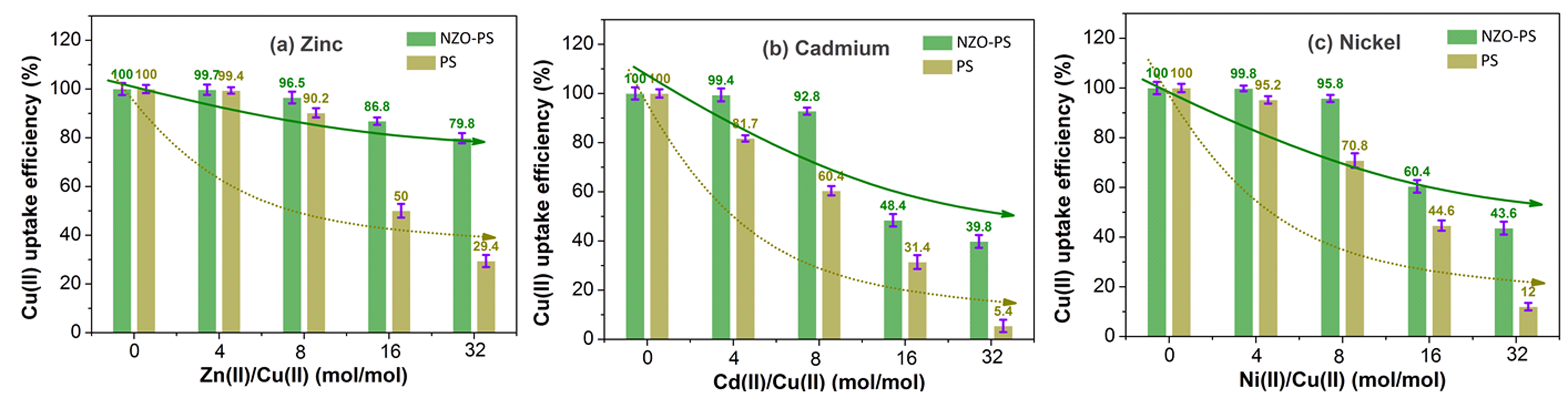
**

**Figure S3** Cu(II) sorption performances onto NZO-PS by various common anions additions

**
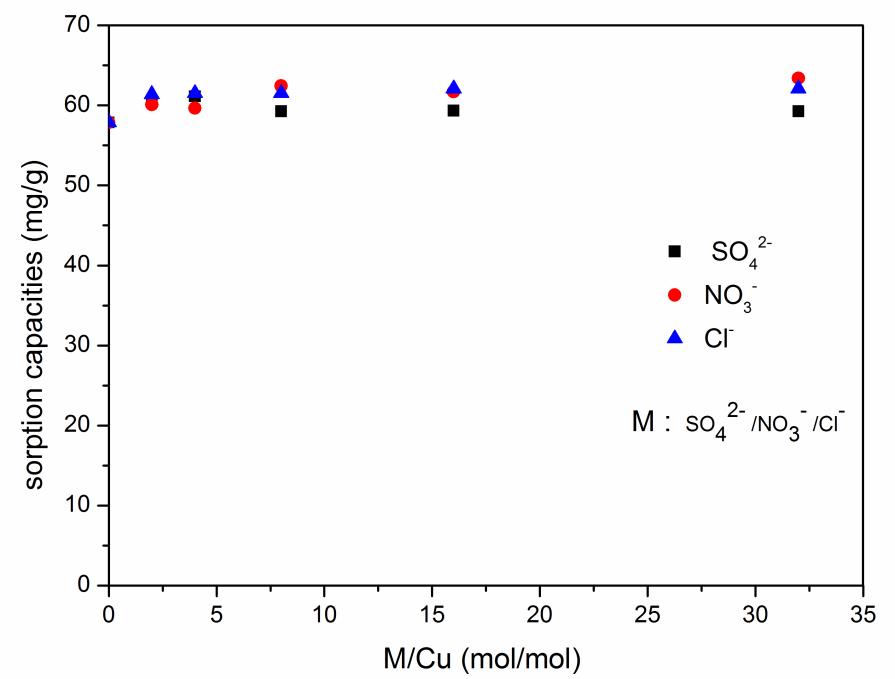
**

**Figure S4** FT-IR spectral analysis. (a) FT-IR spectrum of NZO-PS; (b) ZrO2 samples spectral before and after adsorption of Cu(II) ions.

**
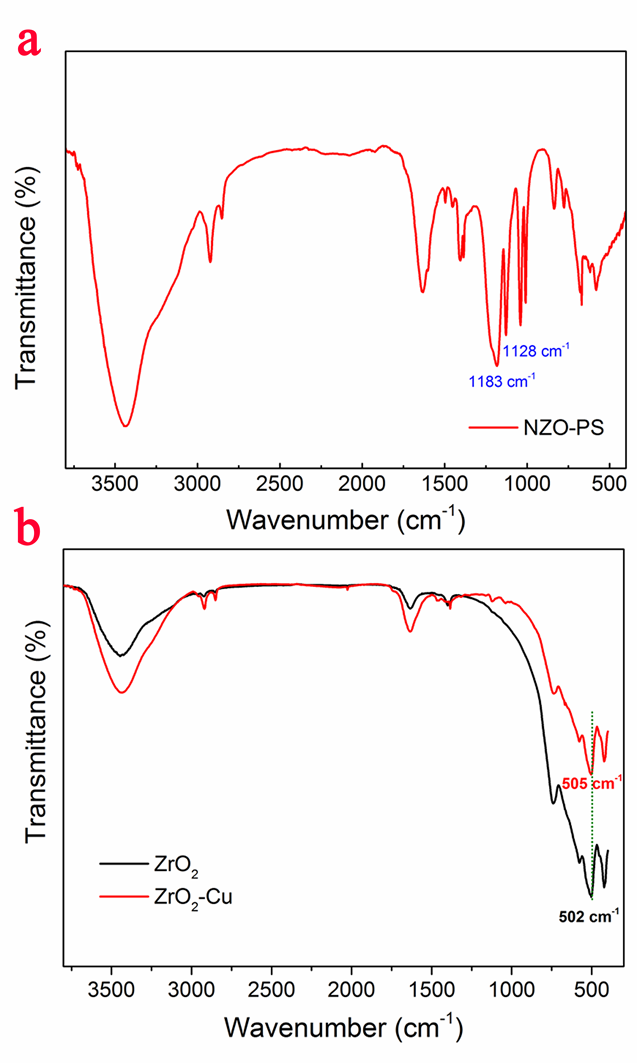
**

**Figure S5** Zeta potentials analysis onto the composite NZO-PS and
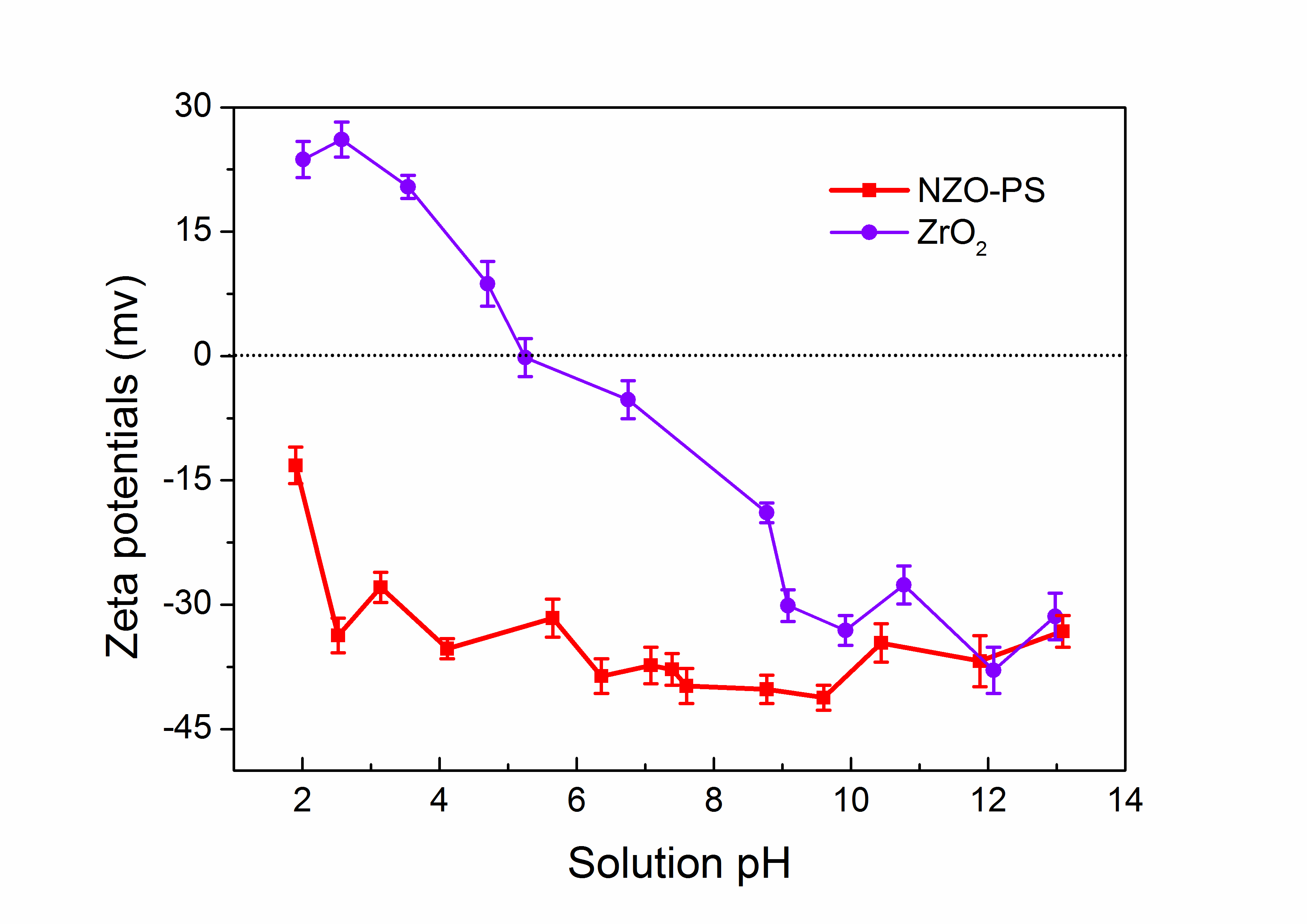
ZrO_2_ particles

**Figure S6** Sorption isotherms onto NZO-PS at different temperatures by Freundlich model fitting.

**
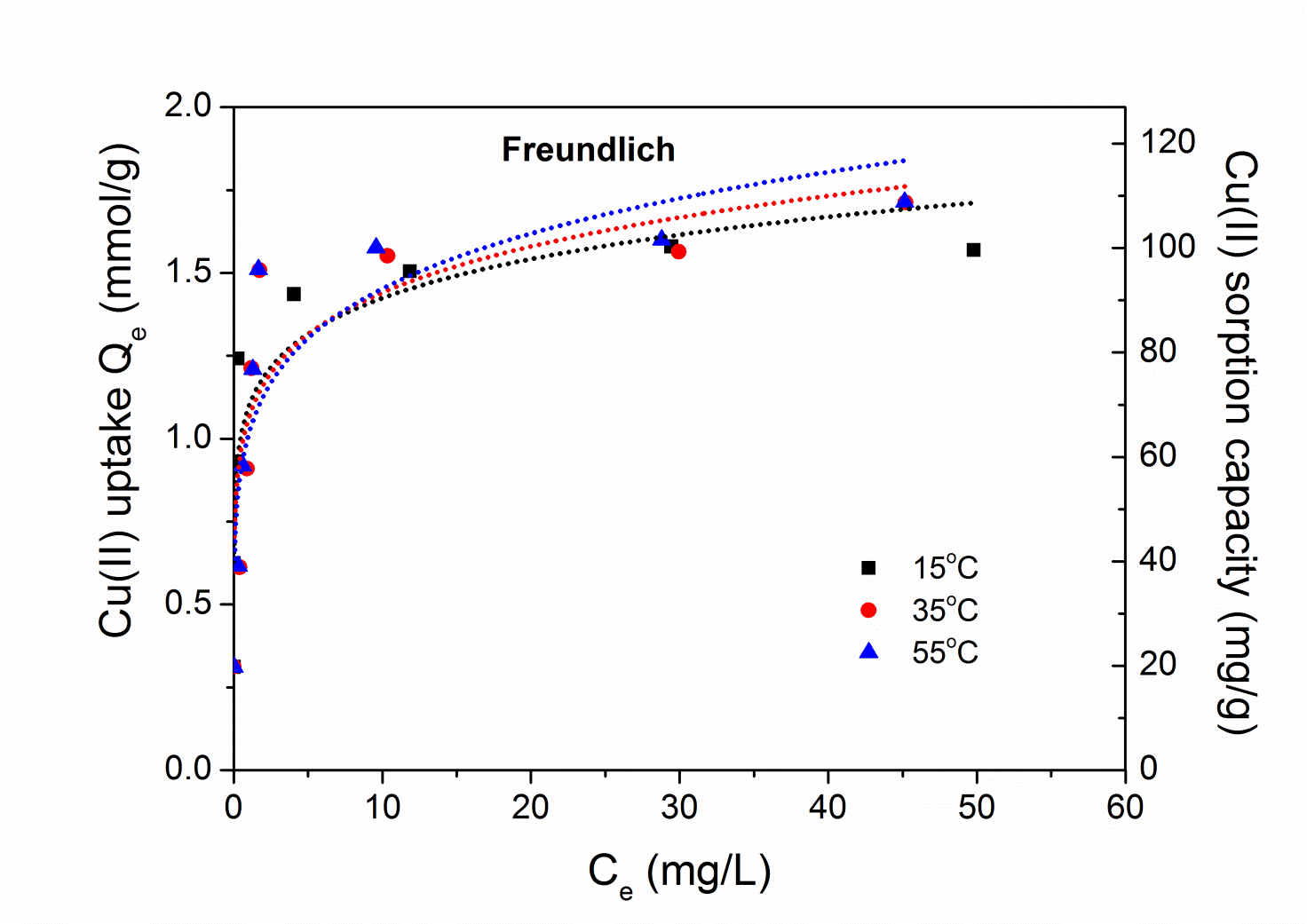
**

**Figure S7** Characterization of NZO-PC, (a) TEM Image of NZO-PC (b) XRD spectrum of NZO-PC


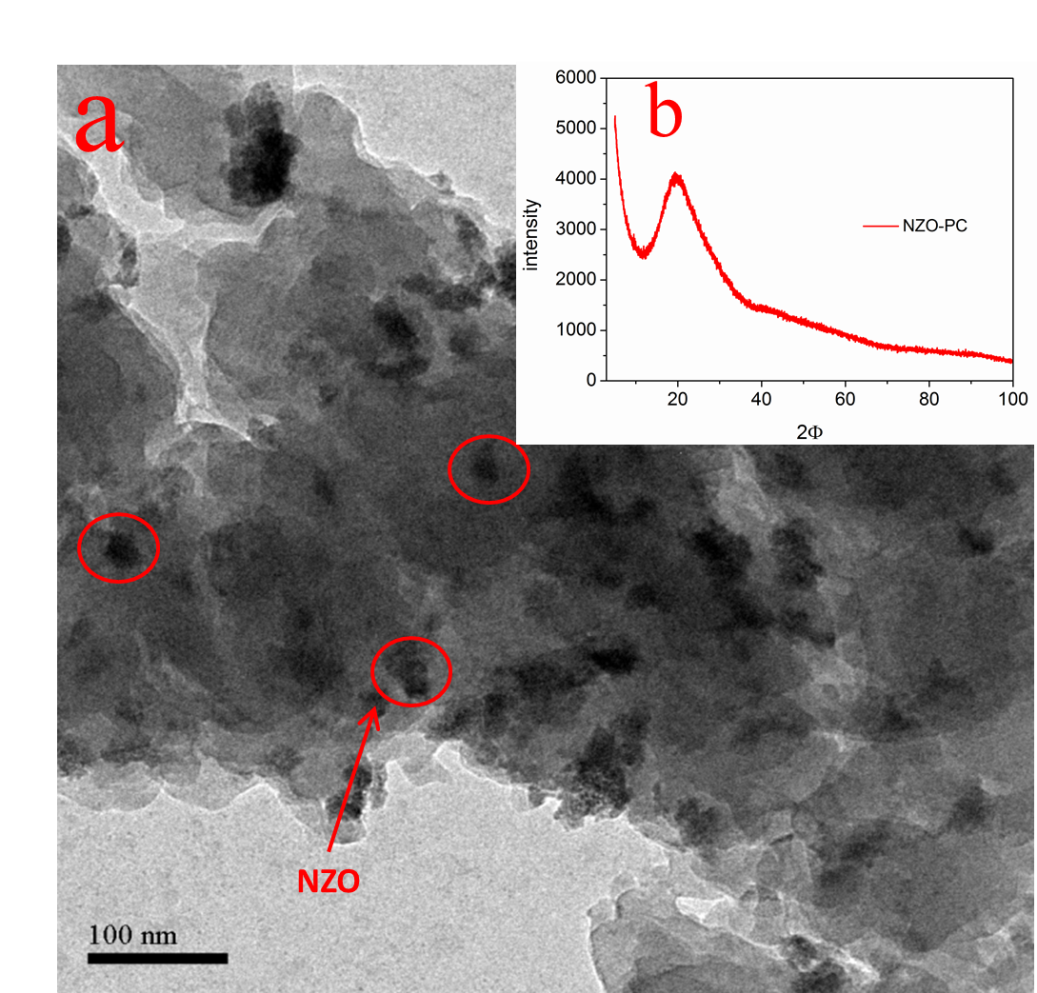


**Figure S8** The box-model scheme illustration of the composited NZO-PS according to the Donnan membrane principle

**
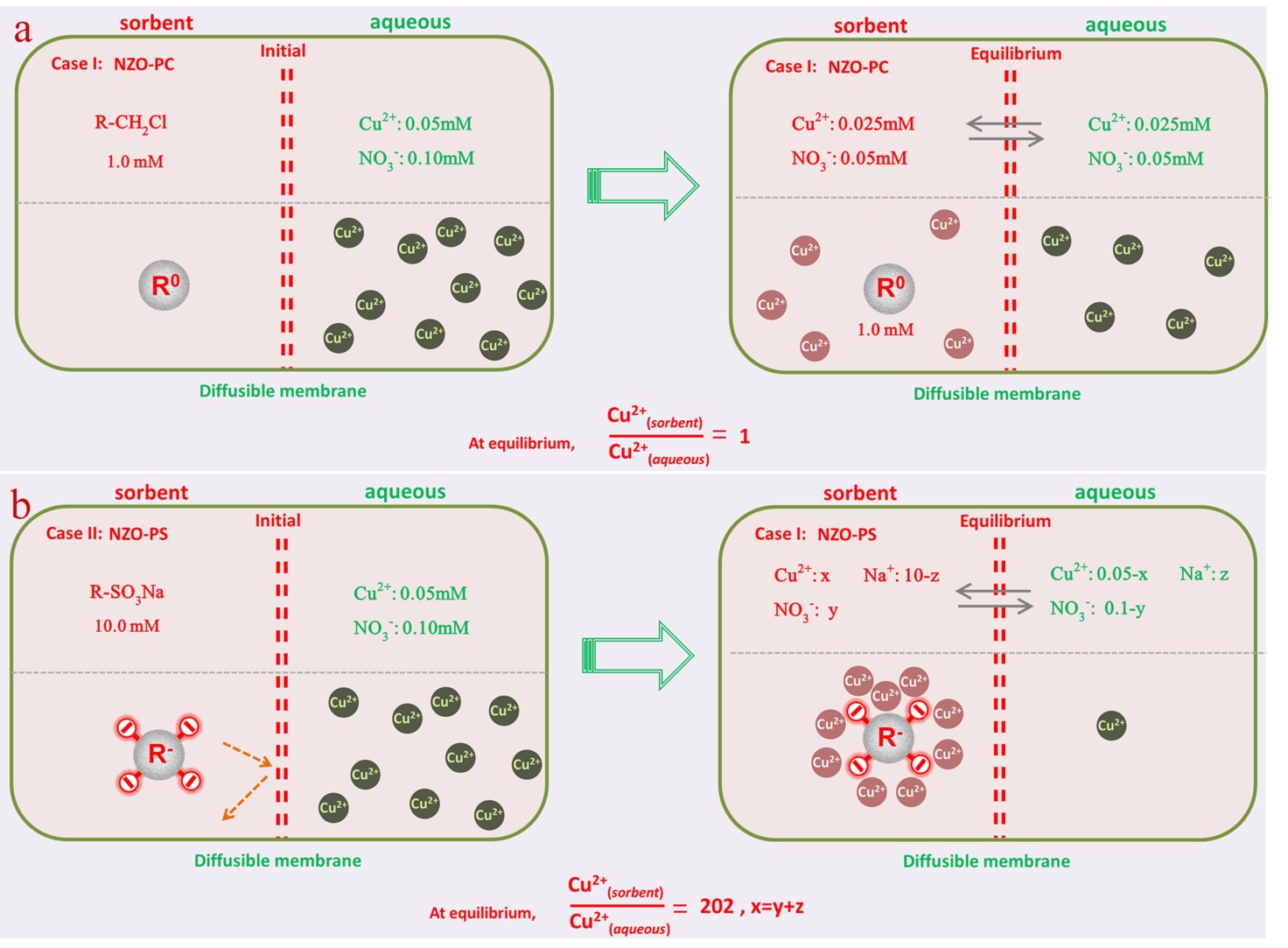
**

**Table S1** Salient properties of NZO-PS, NZO-PC and the host material PS

| Designation | PS | NZO-PS | NZO-PC | ZrO2 |
| --- | --- | --- | --- | --- |
| Matrix structure | Polystyrene | Polystyrene | Polystyrene | NA |
| Surface groups | -SO_3_^-^Na^+^ | -SO_3_^-^Na^+^ | -CH_2_Cl | Zr-OH |
| groups Contents (meq/g) | 4.1 | 3.4 | NA | NA |
| BET surface area (m^2^/g) | 14.8 | 18.3 | 24.4 | 8.35 |
| Average pore diameter (nm) | 23.3 | 14.1 | 14.8 | 6.12 |
| Pore volume (cm^3^/g) | 0.086 | 0.051 | 0.075 | 0.028 |
| ZrO_2_ content ( Zr mass %) | 0 | 9.1 % | 8.4% | 92% |

| **Competing**  **anions(M)** | **Materials** | **K_d_ (L/g) at different initial competing anions (M/Cu mol/mol)** | | | | | |
| --- | --- | --- | --- | --- | --- | --- | --- |
|  |  | 0 | 8 | 16 | 32 | 64 |  |
|  |  |  |  |  |  |  |  |
| Ca(II) | **NZO-PS** | **420** | **4.07** | **1.77** | **1.18** | **0.92** |  |
|  | PS | 503 | 0.358 | 0.208 | 0.103 | 0.070 |  |
| Mg(II) | **NZO-PS** | **420** | **2.91** | **2.03** | **1.81** | **1.52** |  |
|  | PS | 503 | 1.09 | 0.532 | 0.240 | 0.181 |  |
| Na(I) | **NZO-PS** | **420** | **329** | **100** | **36.4** | **10.8** |  |
|  | PS | 503 | 401 | 78.5 | 17.6 | 5.28 |  |
| Zn(II) | **NZO-PS** | **420** | **374** | **27.8** | **6.57** | **3.95** |  |
|  | PS | 503 | 186 | 9.20 | 1.00 | 0.42 |  |
| Cd(II) | **NZO-PS** | **420** | **165** | **12.8** | **0.93** | **0.23** |  |
|  | PS | 503 | 4..47 | 1.52 | 0.65 | 0.06 |  |
| Ni(II) | **NZO-PS** | **420** | **125** | **23.1** | **1.52** | **0.77** |  |
|  | PS | 503 | 20.1 | 2.43 | 0.80 | 0.13 |  |
|  |  | **The K_d_ comparison at various initial Cu(II) contents** | | | | |  |
| **Binary mixtures** | **NZO-PS** | 44.4 | 6.60 | 3.33 | 2.33 | 2.00 |  |
|  | ZrO_2_+ PS | 0.2 | 0.28 | 0.27 | 0.32 | 0.34 |  |

**Table S2**. K_d_ (L/g) Values of Cu(II) adsorption onto NZO-PS , PS and ZrO_2_ + PS mixtures at 298K in the presence of competing cations at different levels.

**Table S3** Kinetic parameters for Cu(II) uptake onto NZO-PS and PS at 298K

| **Materials** | **Pseudo-first-order model** | | | **Pseudo-second-order model** | | | **Intraparticle diffusion model** | |
| --- | --- | --- | --- | --- | --- | --- | --- | --- |
|  | Q_ecal_  (mg/g) | K_1_  (/min) | R^2^ | Q_ecal_  (mg/g) | K_2_  (10^-4^L•min^-1^•mg^-^) | R^2^ | K_p_  (mg/(g.min^0.5^)) | R^2^ |
| NZO-PS | 77.8 | 1.58 | 0.972 | 76.2 | 1.48 | 0.993 | 6.56 | 0.991 |
| PS | 76.9 | 1.52 | 0.995 | 73.7 | 1.43 | 0.997 | 6.45 | 0.990 |

**Table S4** Sorption isotherms parameters for Cu(II) retentions onto NZO-PS at different temperatures

| **Temperature**  **(K)** | **Langmuir model** | | | **Freundlich model** | | |
| --- | --- | --- | --- | --- | --- | --- |
|  | Q_mcal_(mg/g) | K_L_(Lm/mol) | R^2^ | K_F_ | 1/n | R^2^ |
| 288 | 95.8 | 15.2 | 0.900 | 1.09 | 0.114 | 0.785 |
| 308 | 103 | 2.13 | 0.901 | 1.06 | 0.133 | 0.633 |
| 328 | 108 | 2.16 | 0.942 | 1.01 | 0.156 | 0.761 |

**Table S5** Comparison of the Cu(II) sorption capacities of the composite adsorbents

| Adsorbent | Cu(II)  Q_max_(mg/g) | Optimal pH | Temperature  (K) | | Refs | |
| --- | --- | --- | --- | --- | --- | --- |
| loofah fibers | 14.16 | 6.0 | 298K | [^1^](#_ENREF_1) | |  |
| coconut dregs residue | 2.76 | 4-6 | 298K | [^2^](#_ENREF_2) | |  |
| sulfonated magnetic  graphene oxide composite | 63.6 | 5.0 | 323K | [^3^](#_ENREF_3) | |  |
| Spherical polystyrene-supported chitosan thin film | 99.8 | 5.0 | 298K | [^4^](#_ENREF_4) | |  |
| A cellulose-rich biomass | 73.5 | 6.0 | 298K | [^5^](#_ENREF_5) | |  |
| Novel active carbon/crown ether derivative hybrid material | 52.5 | 2.0-7.0 | 298K | [^6^](#_ENREF_6) | |  |
| Porous geopolymeric spheres | 52.6 | 3.0-5.0 | 298K | [^7^](#_ENREF_7) | |  |
| alginate-immobilized bentonite | 114.7 | NA | 298K | [^8^](#_ENREF_8) | |  |
| Novel polyethersulfone nanocomposite membrane by PANI/Fe3O4 nanoparticles | 100.8 | NA | 298K | [^9^](#_ENREF_9) | |  |
| Facial composite adsorbent | 176.2 | 7.0 | NA | [^10^](#_ENREF_10) | |  |
| EBMS immobilization onto mesoporous silica monoliths. | 182.1 | 5.0-7.0 | 298K | [^11^](#_ENREF_11) | |  |
| Modified mesoporous silica monoliths. | 171.5 | 5.0-60. | 298K | [^12^](#_ENREF_12) | |  |
| Ligand modified a new type  mesoporous adsorbent | 145.98 | 6.0-7.0 | NA | [^13^](#_ENREF_13) | |  |
| N,N-bis(salicylidene)1,2-bis(2-aminophenylthio)ethane onto mesoporous silica monoliths | 175.7 | 6.0-7.0 | 298K | [^14^](#_ENREF_14) | |  |
| Conjugate adsorbent | 199.2 | 47.0-5.0 | 298K | [^15^](#_ENREF_15) | |  |
| charged to polystyrene/zirconium oxide nanocomposite | 108 | 5.0-6.0 | 298K | present study | |  |

**Table S6** The main components of electroplating wastewater (unit: mg/L)

| **COD** | **Cu(II)** | |  | **Ni(II)** | | **Zn(II)** | **Ca(II)** | | **Na(I)** | | **SO_4_^2-^** | | **Cl^-^** | | **Salts** | | **pH** |  |
| --- | --- | --- | --- | --- | --- | --- | --- | --- | --- | --- | --- | --- | --- | --- | --- | --- | --- | --- |
| **～280-420** | | ～0.6-0.8 | | ～0.4 | ～1.2 | | ～530 | ～1020 | | ～430 | | ～1490 | | ～4000-7000 | | 7.8-8.4 | | |

**References**

1. Tang, X. et al. Removal of Cu(II) by loofah fibers as a natural and low-cost adsorbent from aqueous solutions. *Journal of Molecular Liquids* **199**, 401-407 (2014).

2. Kamari, A., Yusoff, S.N.M., Abdullah, F. & Putra, W.P. Biosorptive removal of Cu(II), Ni(II) and Pb(II) ions from aqueous solutions using coconut dregs residue: Adsorption and characterisation studies. *Journal of Environmental Chemical Engineering* **2**, 1912-1919 (2014).

3. Hu, X.-j. et al. Removal of Cu(II) ions from aqueous solution using sulfonated magnetic graphene oxide composite. *Separation and Purification Technology* **108**, 189-195 (2013).

4. Jiang, W. et al. Spherical polystyrene-supported chitosan thin film of fast kinetics and high capacity for copper removal. *Journal of Hazardous Materials* **276**, 295-301 (2014).

5. Zhong, Q.-Q., Yue, Q.-Y., Li, Q., Gao, B.-Y. & Xu, X. Removal of Cu(II) and Cr(VI) from wastewater by an amphoteric sorbent based on cellulose-rich biomass. *Carbohydrate Polymers* **111**, 788-796 (2014).

6. Luz Godino-Salido, M. et al. Novel active carbon/crown ether derivative hybrid material for the selective removal of Cu(II) ions: The crucial role of the surface chemical functions. *Chemical Engineering Science* **114**, 94-104 (2014).

7. Ge, Y. et al. Porous geopolymeric spheres for removal of Cu(II) from aqueous solution: Synthesis and evaluation. *Journal of Hazardous Materials* **283**, 244-251 (2015).

8. Tan, W.S. & Ting, A.S.Y. Alginate-immobilized bentonite clay: Adsorption efficacy and reusability for Cu(II) removal from aqueous solution. *Bioresource Technology* **160**, 115-118 (2014).

9. Daraei, P. et al. Novel polyethersulfone nanocomposite membrane prepared by PANI/Fe3O4 nanoparticles with enhanced performance for Cu(II) removal from water. *Journal of Membrane Science* **415–416**, 250-259 (2012).

10. Awual, M.R. A novel facial composite adsorbent for enhanced copper(II) detection and removal from wastewater. *Chemical Engineering Journal* **266**, 368-375 (2015).

11. Awual, M.R. et al. Trace copper(II) ions detection and removal from water using novel ligand modified composite adsorbent. *Chemical Engineering Journal* **222**, 67-76 (2013).

12. Awual, M.R., Rahman, I.M.M., Yaita, T., Khaleque, M.A. & Ferdows, M. pH dependent Cu(II) and Pd(II) ions detection and removal from aqueous media by an efficient mesoporous adsorbent. *Chemical Engineering Journal* **236**, 100-109 (2014).

13. Awual, M.R. et al. Copper(II) ions capturing from water using ligand modified a new type mesoporous adsorbent. *Chemical Engineering Journal* **221**, 322-330 (2013).

14. Awual, M.R., Ismael, M., Khaleque, M.A. & Yaita, T. Ultra-trace copper(II) detection and removal from wastewater using novel meso-adsorbent. *Journal of Industrial and Engineering Chemistry* **20**, 2332-2340 (2014).

15. Awual, M.R., Yaita, T. & Okamoto, Y. A novel ligand based dual conjugate adsorbent for cobalt(II) and copper(II) ions capturing from water. *Sensors and Actuators B: Chemical* **203**, 71-80 (2014).
